# Quick COVID-19 Severity Index, CURB-65 and Quick SOFA Scores Comparison in Predicting Mortality and Risk Factors of COVID-19 Patients

**DOI:** 10.34172/aim.2022.73

**Published:** 2022-07-01

**Authors:** Ayşin Kılınç Toker, İlhami Çelik, İbrahim Toker, Esma Eren

**Affiliations:** ^1^Department of Infectious Disease and Clinical Microbiology, University of Health Sciences Kayseri City Training and Research Hospital, Kayseri, Turkey; ^2^Department of Emergency Medicine, University of Health Sciences Kayseri City Training and Research Hospital, Kayseri, Turkey

**Keywords:** COVID-19, Mortality, Predictive, Risk factors

## Abstract

**Background:**

This study aimed to investigate CURB-65, quick COVID-19 Severity Index (qCSI) and quick Sepsis Related Organ Failure Assessment (qSOFA) scores in predicting mortality and risk factors for death in patients with COVID-19.

**Methods:**

We retrospectively analyzed a total of 1919 cases for whom the rRT-PCR assay for severe acute respiratory syndrome coronavirus 2 (SARS-CoV-2) was positive. For mortality risk factors, univariate and multivariate logistic regression analyses were used. Receiver operator characteristics (ROC) analysis and Kaplan-Meier survival analysis were performed for CURB-65, qCSI and qSOFA scores.

**Results:**

The patients’ average age was 45.7 (21.6) years. Male patients accounted for 51.7% (n=992). In univariate analysis, some clinical variables including age over 65 years and comorbid diseases such as hypertension, chronic kidney disease, malignancy, lymphopenia, troponin, lactate dehydrogenase (LDH) and fibrinogen elevation were associated with the mortality rate. In multivariate logistic regression analysis: Neutrophil lymphocyte ratio (NLR) 3.3 and above (OR, 9.1; 95% CI, 1.9–42), C-reactive protein (CRP)30 mg/L and above (OR, 4.1; 95% CI, 1.2–13.6), D-dimer 1000 ng/mL and above (OR, 4; 95% CI, 1.5–10.7) and age (OR, 1.11; 95% CI, 1.04–1.18-year increase) were identified as risk factors for mortality among COVID-19 patients. The CURB-65 and qCSI scores exhibited a high degree of discrimination in mortality prediction (AUC values were 0.928 and 0.865, respectively). Also, the qSOFA score had a moderate discriminant power (AUC value was 0.754).

**Conclusion:**

CURB-65 and qSCI scores had a high discriminatory power to predict mortality. Also, this study identified CURB-65, qCSI and qSOFA scores, NLR, CRP, D-dimer level, and annual age increase as important mortality risk factors.

## Introduction

 The 2019 coronavirus outbreak (COVID-19) caused by severe acute respiratory syndrome coronavirus 2 (SARS-CoV-2) has led to approximately 105 million confirmed cases and over 2.3 million global deaths.^1^ An estimated 2.5 million patients and 26 000 deaths occurred in Turkey. At the same time, our country is among the countries most affected by the outbreak.^2^

 At the beginning of the pandemic, studies conducted in China showed that severe illness and poor prognosis were associated with comorbid conditions such as hypertension, diabetes, obesity, asthma, chronic obstructive pulmonary disease, or advanced age.^3,4^

 Based on the World Health Organization (WHO) classification, causes of mortality are divided into infectious diseases, non-communicable diseases (NCDs), and injuries.^5^ Cardiovascular diseases, cancers, respiratory diseases and diabetes account for 82% of deaths from NCDs.^6^ The COVID-19 pandemic has become the major reason of death globally due to its infective nature and respiratory effects.

 In many studies, factors predicting severe disease, poor prognosis and mortality have been investigated. Among these factors, extremely advanced age, comorbid conditions and various laboratory parameters, such as the number of white blood cells, neutrophil-lymphocyte ratios, were examined, and valuable information was obtained.^7-9^

 Prognostic risk prediction scores have been developed to facilitate the clinicians’ decision-making processes. The CURB-65 score is a common scale used to estimate the need for hospitalization in adult community-acquired pneumonia. The quick COVID-19 Severity Index (qCSI) is just beginning to be used to predict critical respiratory diseases. It is a simple score using quick Sepsis-Related Organ Failure Assessment (qSOFA) criteria to estimate prognosis in adult patients with suspected sepsis in the out-of-hospital settings, general hospital wards, or emergency departments.^10-12^

 The objective of this study is to evaluate the CURB-65, qCSI and qSOFA scores in predicting mortality and the relationship between clinical and laboratory variables and all-cause mortality in patients who were monitored and treated for COVID-19 in our hospital.

## Materials and Methods

###  Study Design and Patients

 In this study, patients with COVID-19 who were followed up and treated in the Kayseri City Training and Research Hospital between March 13 and May 31, 2020, in Kayseri City Hospital with a 1607 bed capacity and 253 of the intensive care beds were examined.

 The inclusion criteria were patients who had a prior diagnosis of COVID-19 and a positive real-time reverse transcription polymerase chain reaction (rRT -PCR) test on throat samples and nasal swabs.

###  Statistical Analysis

 In the statistical analysis of the study, categorical data were assessed in terms of frequency and percentage. Also, continuous data were evaluated as mean, standard deviation or median (minimum-maximum) based on the data distribution. The Shapiro-Wilk test was used to assess the normality of continuous measurements.

 Descriptive statistics for categorical variables were performed using the chi-square test, while an independent sample *t* test /Mann-Whitney U was used for continuous data.

 Univariate and multivariate logistic regression analyses were applied for mortality risk factors. Risk factors that were significant in univariate analysis were analyzed using multivariate binary logistic regression. The crude and adjusted odds ratios (ORs) and 95% confidence intervals (CIs) were estimated.

 The Kaplan-Meier survival test was employed to predict one-month post-admission mortality based on the CURB-65, qCSI, and qSOFA scores.

 The level of significance (*P* value) was considered to be 0.05. The receiver operator characteristics (ROC) analysis was conducted for the qSOFA, qCSI and CURB-65 scores. Also, the ROC curves for these parameters were compared. The area under the curve (AUC), sensitivity, specificity, positive predictive value (PPV), negative predictive value (NPV), positive likelihood ratio (LR + ) and negative likelihood ratio (LR-) values were given as descriptive statistics.

###  Definitions

*Quick SOFA criteria ( qSOFA ):*The qSOFA criteria determine the prognosis induced by sepsis in adult patients with suspected infections in nonhospital, emergency or general hospital conditions. A positive qSOFA score needs two or more of those items: breathing rate 22 breaths/minute or more, altered mental status or systolic blood pressure less than 100 mm Hg.^10^

*CURB-65 score:* The CURB-65 score (Confusion, Urea > 19 mg/dL, Respiratory rate > 30/min, low systolic ( < 90 mm Hg) or diastolic ( < 60 mm Hg) blood pressure, age > 65 years) is used to determine the need for hospitalization in adults diagnosed with community-acquired pneumonia. The CURB-65 score varies from 0 to 5. Marks 0-1 indicate a low mortality risk, while a mark of at least 2 is associated with higher mortality.^11^

*COVID-19 Rapid Severity Index ( qCSI ):*The quick COVID-19 Severity Index score predicts the 24-hour risk of severe respiratory illness among patients admitted to ED with COVID-19. The qCSI is a 12-point scale that uses only three bedside variables: nasal cannula flow rate, respiratory rate, and documented minimal pulse oximetry. The patients are then divided into four risk strata (0–3) based on the following scores: 0–3 low risk, 4–6 low-medium risk, 7–9 high-medium risk and ≥ 10 high risk.^12^

## Results

 Between March 13 and May 31, 2020, 4,103 patients were admitted to our hospital’s pandemic department for a prior diagnosis of COVID-19. Of these, 1919 patients tested positive for rRT-PCR, and all of these patients were included in the study.

 The mean age of the patients was 45.7 ( ± 21.6) years, and 51.7% (n = 992) of the patients were male. While 86.2% (n = 1654) of the patients were hospitalized in the isolation ward, 13.8% (n = 265) were followed in the intensive care unit. The average length of stay in the hospital for patients was nine days (IQRs, 6–11).

 The most frequent features were cough (44.3%), fever (33.5%), shortness of breath (26.9%), weakness (24.7%) and myalgia (18.9%). However, 16.2% (n = 310) of the patients had symptoms such as loss of taste, smell, and diarrhea. Nausea/vomiting (6.8%) and headache (6.1%) were less common symptoms; 14.5% (n = 279) of patients had a comorbidity, and 13.6% (n = 261) of patients had more than one other condition. The most frequent concomitant diseases were hypertension (HT, 15.7%), diabetes mellitus (DM, 11.2%), coronary heart disease (CAD, 7.3%) and chronic obstructive pulmonary disease (COPD, 7.2%).

 There was no significant difference between genders in terms of factors affecting mortality. While the median age of non-survivors was 74.9 ± 11.2 years, that of survivors was 43.5 ± 20.6 years, which was statistically significant (*P* < 0.001). The age group with the highest mortality rate was between 75 and 84 years of age and showed a significant difference of 33.1% among the other age groups (*P* < 0.001) (Table 1).

**Table 1:** Comparison of Basic Characteristics of Patients with COVID-19 According to Mortality

**Characteristics**	**Total (n=1919)**	**Death (n=130)**	**Survive (n=1789)**	***P*** ** Value**
Age (y), mean ± SD	45.7 ± 21.6	74.9 ± 11.2	43.5 ± 20.6	< 0.001^*^
Age (y), Range, No. (%)				
< 65	1490 (77.6)	26 (20)	1464 (81.8)	< 0.001
65–74	222 (11.6)	33 (25.4)	189 (10.6)	
75–84	152 (7.9)	43 (33.1)	109 (6.1)	
≥ 85	55 (2.9)	28 (21.5)	27 (1.5)	
Gender, No. (%)				
Female	927 (48.3)	62 (47.7)	865 (48.4)	0.885
Male	992 (51.7)	68 (52.3)	924 (51.6)	
Comorbidities, No. (%)				
Hypertension	302 (15.7)	43 (33.1)	259 (14.5)	< 0.001
Diabetes	214 (11.2)	30 (23.1)	184 (10.3)	< 0.001
COPD	138 (7.2)	22 (16.9)	116 (6.5)	< 0.001
Cardiovascular disease	140 (7.3)	27 (20.8)	113 (6.3)	< 0.001
Chronic kidney disease	71 (3.7)	29 (22.3)	42 (2.3)	< 0.001
Malignancy	27 (0.7)	15 (11.5)	12 (0.7)	< 0.001
CT findings, No. (%)				
Negative/normal	504 (29.9)	7 (5.5)	497 (31.9)	< 0.001
Typical	916 (54.4)	72 (56.7)	844 (54.2)	
Indeterminate	173 (10.3)	36 (28.3)	137 (8.8)	
Atypical	92 (5.5)	12 (9.4)	80 (5.1)	
CURB-65 score, No. (%)				
0 or 1	1582 (82.2)	3 (2.3)	1579 (88.3)	< 0.001
2	159 (8.3)	28 (21.5)	131 (7.3)	
≥ 3	178 (9.3)	99 (76.2)	79 (4.4)	
qCSI score, No. (%)				
≤ 3	415 (40.1)	1 (0.8)	414 (45.7)	< 0.001
4–6	362 (35)	34 (26.2)	328 (36.2)	
7–9	110 (10.6)	6 (4.6)	104 (11.5)	
10–12	148 (14.3)	89 (68.5)	59 (6.5)	
qSOFA criteria, No. (%)				
< 2	674 (42.2)	43 (33.1)	631 (71.7)	< 0.001
≥ 2	336 (57.8)	87 (66.9)	249 (28.3)	
Length of stay in hospital, median IQRs, day	9 (IQRs: 6-11)	9 (IQRs: 6-11)	7 (IQRs: 3-14)	0.116^**^
**Laboratory Findings**				
WBC, No. (%)				
≤ 4	202 (10.7)	5 (3.9)	197 (11.2)	< 0.001
4–10	1396 (74)	53 (41.4)	1343 (76.4)	
> 10	289 (15.3)	70 (54.7)	219 (12.5)	
Lymphocyte count, No. (%)				
≥ 1.1	1460 (77.4)	54 (42.2)	1406 (79.9)	< 0.001
< 1.1	427 (22.6)	74 (57.8)	353 (20.1)	
NLR, No. (%)				
< 3.3	1262 (66.9)	13 (10.2)	1249 (71)	< 0.001
≥ 3.3	625 (33.1)	115 (89.8)	510 (29)	
CRP, No. (%)				
< 30	1383 (72.9)	25 (19.2)	1358 (76.8)	< 0.001
≥ 30	515 (27.1)	105 (80.8)	410 (23.2)	
Procalcitonin, No. (%)				
< 0.05	721 (46.8)	706 (49.5)	15 (13.3)	< 0.001
≥ 0.05	819 (53.2)	98 (86.7)	721 (50.5)	
LDH, No. (%)				
≤ 245	666 (56.6)	25 (22.7)	641 (60.1)	< 0.001
> 245	511 (43.4)	85 (77.3)	426 (39.9)	
D-dimer, No. (%)				
< 1000	752 (80)	18 (24.7)	734 (84.7)	< 0.001
≥ 1000	188 (20)	55 (75.3)	133 (15.3)	
Fibrinogen, No. (%)				
Normal	737 (53.4)	45 (42.5)	692 (54.4)	0.018
High	642 (46.6)	61 (57.5)	581 (45.6)	
cTnI (Troponin I), No. (%)				
< 0.3	1275 (96.7)	90 (88.2)	1185 (97.4)	< 0.001
≥ 0.3	44 (3.3)	12 (11.8)	32 (2.6)	

COPD, chronic obstructive pulmonary disease; CT, computed tomography; qCSI, quick COVID-19 Severity Index; qSOFA, quick Sepsis Related Organ Failure Assessment; IQR, Interquartile range; WBC, white blood count; CRP, C-reactive protein; NLR, neutrophil to lymphocyte ratio; LDH, lactate dehydrogenase. ^*^ *P* = Student’s t-test. ^**^ *P* = Mann- Whitney U test. Other *P* values calculated by chi-square test.

 Presence of comorbid diseases, chest tomography findings typical for COVID-19, leukocytosis, lymphopenia, neutrophil-lymphocyte ratio (NLR) equal to or above 3.3, elevation of C-reactive protein (CRP), D-dimer, procalcitonin, lactate dehydrogenase (LDH), Fibrinogen and Troponin were factors associated with higher mortality rate (*P* < 0.001) (Table 1).

 The results of multivariable regression analysis are presented in Table 2.

**Table 2:** Risk Factors of Mortality for COVID-19 Patients

**Risk Factors**	**Univariate OR (95% CI)**	***P*** ** Value**	**Multivariate OR (95% CI)**	***P*** ** Value**
Age (y)	1.109 (1.09–1.127)	< 0.001	1.11 (1.04–1.18)	0.001
Age, > 65 y vs < 65 y	4.4 (3.8- 5)	< 0.001	—	
Female vs male	—	0.814	—	—
Comorbidities				
Hypertension	2.2 (1.7- 2.9)	< 0.001	—	—
Diabetes	2.2 (1.5- 3.1)	< 0.001	—	—
COPD	2.6 (1.7- 3.9)	< 0.001	—	—
CVD	3.2 (2.2- 4.8)	< 0.001	—	—
CKD	9.5 (6.1- 14.7)	< 0.001	—	—
Malignancy	17.2 (8.2- 35.9)	< 0.001	—	—
WBC				
≤ 4 (ref)				
4–10	—	—	—	—
> 10	12.5 (4.9- 31.8)	< 0.001	—	—
Lymphocyte count				
≥ 1.1 (ref)				
< 1.1	2.8 (2.4- 3.4)	< 0.001	—	—
NLR				
< 3.3 (ref)				
≥ 3.3	3.09 (2.8- 3.4)	< 0.001	9.1 (1.9-42)	0.005
CRP				
< 30 (ref)				
≥ 30	3.4 (3- 3.9)	< 0.001	4.1 (1.2- 13.6)	0.017
Procalcitonin				
< 0.05 (ref)				
≥ 0.05	1.7 (1.5- 1.8)	< 0.001	—	—
LDH				
≤ 245 (ref)				
> 245	1.9 (1.6- 4.3)	< 0.001	—	—
D-dimer				
< 1000 (ref)				
≥ 1000	4.9 (4- 6)	< 0.001	4 (1.5- 10.7)	0.006
Fibrinogen				
Normal (ref)				
High	1.2 (1- 1.5)	0.018	—	—
cTnI (Troponin I)				
< 0.3 (ref)				
≥ 0.3	4.4 (2.3- 8.4)	< 0.001	—	—
CURB-65 score	5.6 (4.5- 6.9)	< 0.001	3.9 (2.9- 5.2)	< 0.001
qCSI score	1.5 (1.4- 1.6)	< 0.001	1.3 (1.2- 1.4)	< 0.001
qSOFA criteria				
< 2 (ref)				
≥ 2	5.1 (3.4- 7.6)	< 0.001	2.2 (1.1- 4.1)	0.014

OR, odds ratio; COPD, chronic obstructive pulmonary disease; CKD, *chronic kidney disease; CVD, cardiovascular disease; *qCSI, quick COVID-19 Severity Index; qSOFA, quick Sepsis Related Organ Failure Assessment; WBC, white blood count; CRP, C-reactive protein; NLR, neutrophil to lymphocyte ratio; LDH, lactate dehydrogenase.

 The CURB-65 (OR, 3.9; 95% CI, 2.9–5.2-point increase), the qCSI score (OR, 1.3; 95% CI, 1.2–1.4-point increase) and qSOFA criteria ≥ 2 (OR, 2.2 95% CI, 1.1–4.1) were identified as risk factors for mortality among COVID-19 patients.

 Also, NLR ≥ 3.3 (OR, 9.1; 95% CI, 1.9–42), CRP ≥ 30 mg/L (OR, 4.1; 95% CI, 1.2-13.6), D-dimer ≥ 1000 ng/mL (OR, 4; 95% CI, 1.5–10.7) and age (OR, 1.11; 95% CI, 1.04–1.18-year increase) were identified as risk factors for mortality among COVID-19 patients.

 The mortality rate for COVID-19 was associated with age over 65 and comorbid diseases (HT, DM, CAD, COPD, chronic renal disease and malignancy). However, it proved negligible in multivariate analysis. Similarly, lymphopenia, high levels of LDH, fibrinogen and troponin were factors associated with the COVID-19 mortality rate but were not significant in the multivariate comparison (Table 2).

**Figure 1:**
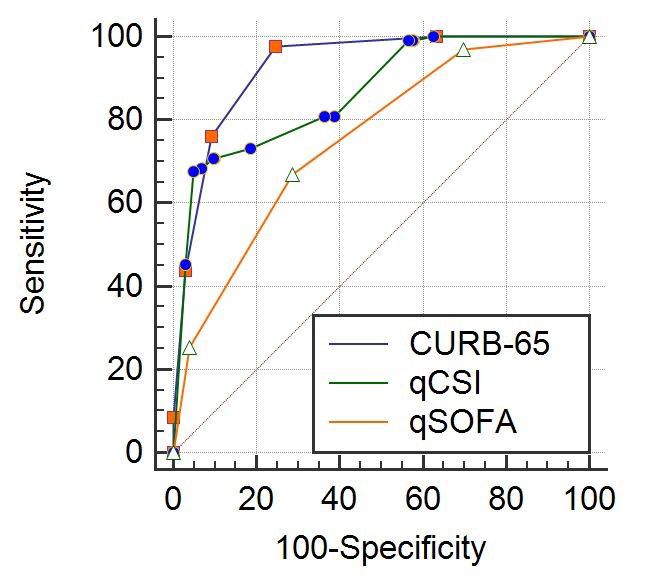
Comparison of ROC Curves for qCSI, CURB-65 and qSOFA

 The CURB-65 and qCSI scores were highly discriminatory in mortality prediction (*P* values ≤ 0.001, AUC values were 0.928 and 0.865, respectively). Also, the qSOFA score had moderate discriminant power in the prediction of mortality (*P* value ≤ 0.001, AUC value was 0.754). Besides, the CURB-65 and qCSI scores were more capable of predicting death than the qSOFA scores. The CURB-65 score greater than 1 had a sensitivity of 97.7%, a specificity of 88.2%, a positive predictive value of 37.7%, a negative predictive value of 99.8%, a positive likelihood ratio of 8.3, and a negative likelihood ratio of 0.026 for predicting death. In addition, the qCSI score greater than 10, had a sensitivity of 67.7%, a specificity of 95.3%, PPV of 67.7%, NPV of 95.4%, LR + of 14.6, and LR- of 0.34 for predicting death (Table 3, Figure 1).

**Table 3:** Comparison of Scores’ Ability to Predict Death

**Scores**	**Cut-off**	**AUC (P Value)**	**Sensitivity (%)**	**Specificity (%)**	**PPV (%)**	**NPV (%)**	**LR+**	**LR-**
CURB-65	> 1	0.928 ( < 0.001)	97.7	88.2	37.7	99.8	8.3	0.026
qCSI	> 10	0.865 ( < 0.001)	67.7	95.3	67.7	95.4	14.6	0.34
qSOFA	> 1	0.754 ( < 0.001)	66.9	71.7	25.9	93.6	2.37	0.46

AUC, Area under the ROC curve; PPV, positive predictive value; NPV, negative predictive value; LR + , positive likelihood ratio; LR-, negative likelihood ratio.

**Figure 2:**
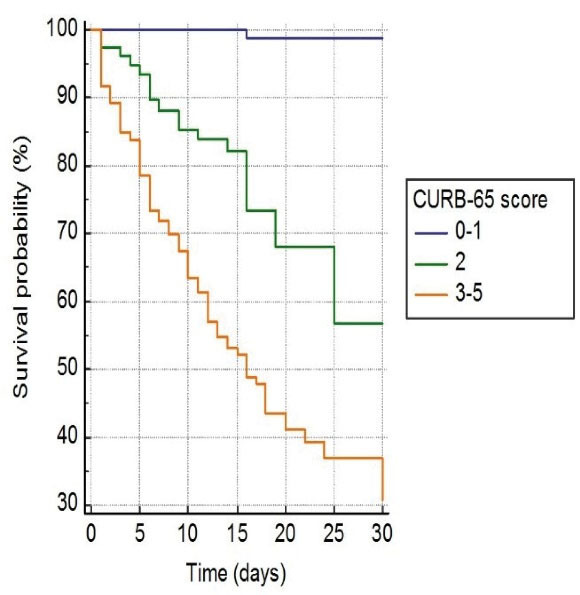
Kaplan-Meier Curve for One-Month Fatality among COVID-19 Patients Based on CURB-65 Score on Admission

**Figure 3:**
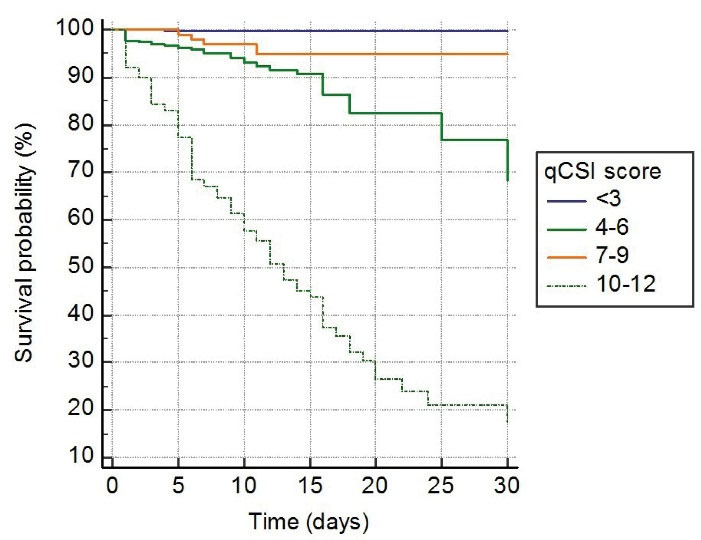
Kaplan-Meier Curve for One-Month Fatality among COVID-19 Patients Based on qCSI Score on Admission

**Figure 4:**
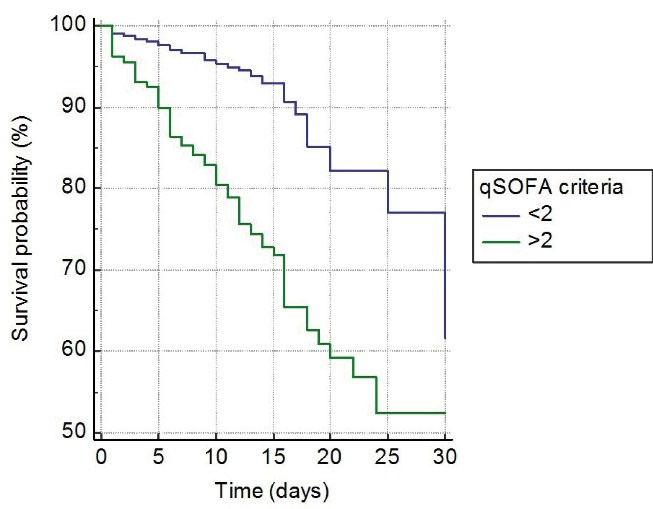
Kaplan-Meier Curve for One-Month Fatality among COVID-19 Patients Based on qSOFA Score on Admission

 In survival analysis, of 122 (6.3%) patients who died during one-month follow-up, 2 (0.1%) patients had 0 to 1 point, 27 patients (16.9%) had 2 points, and 93 (52.2%) patients had 3 to 5 points on CURB-65 (*P* < 0.001, Figure 2). Similarly, of 122 patients who died, one (0.2%) patient had < 3 points, 32 (8.8%) patient had 4 to 6 points, 4 (3.6%) patient had 7 to 9 points, and 85 (57.4%) had 10 to 12 points on qCSI (p < 0.001, Figure 3). In addition, of 122 patients who died, 39 (5.7%) had < 2 points, and 83 (24.7%) had > 2 points on qSOFA (*P* < 0.001, Figure 4).

## Discussion

 In our research, CURB-65 and qCSI scores showed a strong power of discrimination in predicting mortality. Moreover, the qSOFA score had enough discriminatory ability to predict mortality. And we assessed whether NLR ≥ 3.3, CRP ≥ 30 mg/L, and D-Dimer ≥ 1000 ng/mL were significant risk factors associated with mortality.

 Mortality due to COVID-19 was 15% during the early phases of the pandemic. However, with the increase in caseload, the rate remained between 4.3% and 11%. According to recent data, it has declined to 3.4%.^13^ We found a 6.8% mortality rate in our study.

 Aging and increasing co-morbidities are independent predictors of inpatient fatality amongst COVID-19 patients.^7,14-16^ Elderly patients have a weaker immune response due to many different pathophysiological mechanisms, such as a decrease in the number of innate immune cells, recognition of pathogen-associated molecular patterns (PAMPs), cytokine regulation, pure lymphocyte/memory lymphocyte ratio, a reduction in the circulating plasma cell number and a predominance of Th2 to Th1 responses. COVID-19 is more severe in older adults. The decrease in immune response with aging is defined as immunosenescence. The aging of hematopoietic stem cells and deterioration in immune system cells’ number, function, and activity are essential causes of immunosenescence. For these reasons, there may be higher SARS-CoV-2 replication in older people.^17^

 Elevated white blood count (WBC) has been a poor prognostic factor in many studies. Research published in the literature shows that WBC accounts are increased in patients with severe COVID-19 compared to mild cases. However, studies show that normal or decreased WBC counts are more prevalent among COVID-19 patients than healthy individuals.^18^

 While neutrophils represent the innate immune response, lymphocytes are the marker of the inflammatory response. Thus, high NLR indicates an instability in the inflammatory response in sepsis and bacteremia. NLR is reliable in the prognosis of viral diseases.^8^ A meta-analysis of 15 studies showed that patients with severe COVID-19 had elevated neutrophil and NLR levels despite a low number of lymphocytes. Increased expression of natural killers and T-cell immunoglobulins is associated with T lymphocytes’ functional depletion at the early stage of viral infections. One possible cause of this finding is the exhaustion of lymphocytes during ingestion of the virus. Because the angiotensin-converting enzyme two receptors are expressed in lymphocytes, SARS-CoV-2 directly infects and destroys lymphocytes. This situation plays a role in the pathogenesis of COVID-19. Studies have demonstrated the predictive value of NLR and its relation to mortality.^8,19^ Our study similarly showed that the increased rate of NLR is a significant risk factor associated with mortality.

 CRP, an acute-phase protein, is one of the most commonly used biomarkers to demonstrate infection and inflammation. The higher CRP level in the blood plays an informative role in the immune response acquired as a recognized innate lectin, originally a pentameric polypeptide. Elevated serum PRC rates have been demonstrated in most COVID-19 patients and have been associated with mortality. It has also been shown that CRP at the time of admission is associated with disease severity in COVID 19 patients. It may also be a powerful predictor of death.^20-22^ A possible explanation for the increase in CRP in COVID-19 could be the excessive production of inflammatory cytokines. When the immune system is overactive, the increased cytokine response to pathogens can damage the lung tissue. CRP production is induced as a result of excess cytokine production and tissue destruction.^23^

 Increased mortality was reported to be related to the 1000 ng/mL D-dimer concentration in COVID-19 patients.^14^ D-dimer levels are commonly elevated in COVID-19 patients, and patients with high D-dimers have worse clinical outcomes (all-cause mortality, ICU hospitalization, or acute respiratory distress syndrome). It has been shown that D-dimer measurement can guide clinical decision-making and can be a reliable prognostic marker.^24,25^ Possible reasons why D-dimmer augmentation is associated with poor clinical outcomes include: 1) Patients suffering from severe COVID-19 may have diffuse intravascular coagulation secondary to septicaemia; 2) A severe acute respiratory infection can damage endothelial cells and enhance haemostatic factors such as D-dimer and von Willebrand factor; 3) Respiratory tract infections such as H1N1 influenza can cause the possible formation of pulmonary microthrombus and, consequently, raise D-dimer level; 4) It is assumed that patients with severe COVID 19 may be more likely to have additional complications such as acute kidney injury, critical heart damage, congestive heart failure, all of which may lead to increased D-dimer levels.^24^

 Our study assessed the performance of CURB-65, qCSI and qSOFA scores to predict mortality among COVID-19 patients. CURB-65 and qSOFA are well-known predictive testing and risk stratification tools. The 3-variable (respiratory rate, pulse oximetry and oxygen flow rate) qCSI score was better than the CURB-65 and qSOFA score in the independent validation cohort. The qCSI score development validation study was completed on a total of 1172 patients. In this study, a qCSI index greater than 9 had a specificity of 99% and + LR 8.36 for predicting respiratory failure.^26^ In another study on 313 consecutive adult patients, the CURB-65 and qSCI scores were good for estimating fatality. In this study, CURB-65 (AUC 0.781) was superior to qCSI (AUC 0.711) for predicting hospital death.^27^ In our research, the CURB-65 and qCSI scores had a high discriminatory effect on mortality prediction (the AUC values were 0.928 and 0.865, respectively). Moreover, the qSOFA score was sufficiently discriminatory to predict mortality (the CSA value was 0.754).

 Our study’s primary limitations are that it is retrospective, that the survey duration was short, and that it included only inpatients. Secondly, the study was carried out in only one tertiary care center. Consequently, this study may include patients with disproportionately worse outcomes.

 In conclusion, in this study, CURB-65 and qCSI scores had a strong discriminatory effect in predicting mortality. Also, CURB-65, qCSI and qSOFA scores, NLR, CRP, D-dimer levels, and the annual increase in age were significant risk factors associated with mortality.
